# Validation of the Turkish version of the Sunnybrook facial grading system

**DOI:** 10.3906/sag-1905-195

**Published:** 2020-04-09

**Authors:** Erdem MENGİ, Cüneyt Orhan KARA, Fazıl Necdet ARDIÇ, Funda TÜMKAYA, Fevzi BARLAY, Taylan ÇİL, Hande ŞENOL

**Affiliations:** 1 Department of Otorhinolaryngology, Faculty of Medicine, Pamukkale University, Denizli Turkey; 2 Department of Biostatistics, Faculty of Medicine, Pamukkale University, Denizli Turkey

**Keywords:** Facial nerve, facial palsy, Sunnybrook facial grading system, validation

## Abstract

**Background/aim:**

To adapt the Sunnybrook facial grading system (SFGS) into Turkish and perform validation and reliability studies on the Turkish version.

**Materials and Methods:**

The original English version of the SFGS was translated into Turkish by performing a linguistic validity study based on international standards. The evaluators comprised 6 physicians. Evaluations were performed twice independently using the video recordings of 65 facial palsy patients. Synchronously, the House-Brackman facial grading system (HBFGS) was filled out to display concurrent validity. The intraclass correlation coefficient (ICC) and Cronbach’s alpha was used for the examination of the inter- and intra-rater reliability. As another indication of reliability, the generalizability (G) was also examined.

**Results:**

The ICC for the inter-rater reliability for resting symmetry, symmetry of voluntary movement, synkinesis, and the composite score, which are 4 components of the SFGS, were determined, respectively, as 0.822, 0.956, 0.606, and 0.957 for the first evaluation, and 0.805, 0.965, 0.584, and 0.965 for the second evaluation. For the intra-rater reliability, the ICC were determined as 0.842, 0.956, 0.794, and 0.937, while the Cronbach’s alpha coefficients were determined as 0.809, 0.956, 0.792, and 0.948, respectively. The G coefficient was determined as G = 0.772. For the concurrent validity, a strong correlation was found between the SFGS and HBFGS scores.

**Conclusion:**

The present study adapted the SFGS into Turkish, and demonstrated that the adapted scale was valid and reliable. The Turkish version can be used for the evaluation of facial palsy, the follow-up of treatment efficiency, and standardization in reporting outcomes with the international literature.

## 1. Introduction

Several grading systems have been developed to evaluate the severity of paralysis for peripheral facial palsy (PFP) patients. These grading systems are based on the observations of the physicians who examine the patient. These systems are quite important for the correct and standard evaluation of diagnosis and therapy. 

The system that is currently the most commonly used is the House-Brackman facial grading system (HBFGS). It was defined in 1985 by the Facial Nerve Disorders Committee of the American Academy [1]. It is known that this system, in which the patients are graded from 1 to 6 according to the severity of the facial functions, has numerous criticisms, such as evaluation of the upper and lower parts of the face in the same grade, the overlap of facial movements between grades, and not being sensitive enough to clinical changes in facial functions [2,3]. Due to these criticisms, alternative clinical grading systems have been suggested over time. The Sunnybrook facial grading system (SFGS) is one of the most widely accepted systems in the literature due to its reliability and reproducibility [4]. In a multicentered systematic review about facial nerve grading instruments, it was indicated that the SFGS, which is among the 19 facial grading systems defined to date, is the only system that meets all of the criteria on this topic, and it was proposed that it should be used as the standard grading system worldwide [5]. However, there has been no validated Turkish version of this evaluation system. 

In this study, it was aimed to translate the SFGS, which has been gradually more accepted around the world, into Turkish and perform validity and reliability studies on the Turkish version of the SFGS.

## 2. Materials and methods

### 2.1. Turkish adaptation stage

In this study, written permission was obtained from Ross et al., the authors of the original English SFGS, for the Turkish adaptation [4]. After permission was granted, the linguistic validity of the adapted scale in Turkish was evaluated. At this stage, the original test text was translated to Turkish by 2 researchers independently of one another. Later, these 2 researchers came together and transformed the test to a single translated text. This Turkish text was then translated back into English by another researcher who was experienced in the field of otology. Afterwards, the original text, and the Turkish and English translated texts were examined by these 3 researchers. The final version of the Turkish text was determined via discussion of the differences. The researchers then agreed on the final Turkish text to be used. The language validation study was concluded at this point, since the system is a technical text. After completion of the language adaptation process, the Turkish system was structured properly to the original system (Figure). Prior to administration of the system, ethical committee approval for the research was obtained from the Pamukkale University Ethical Committee (60116787-020/4322). 

**Figure:**
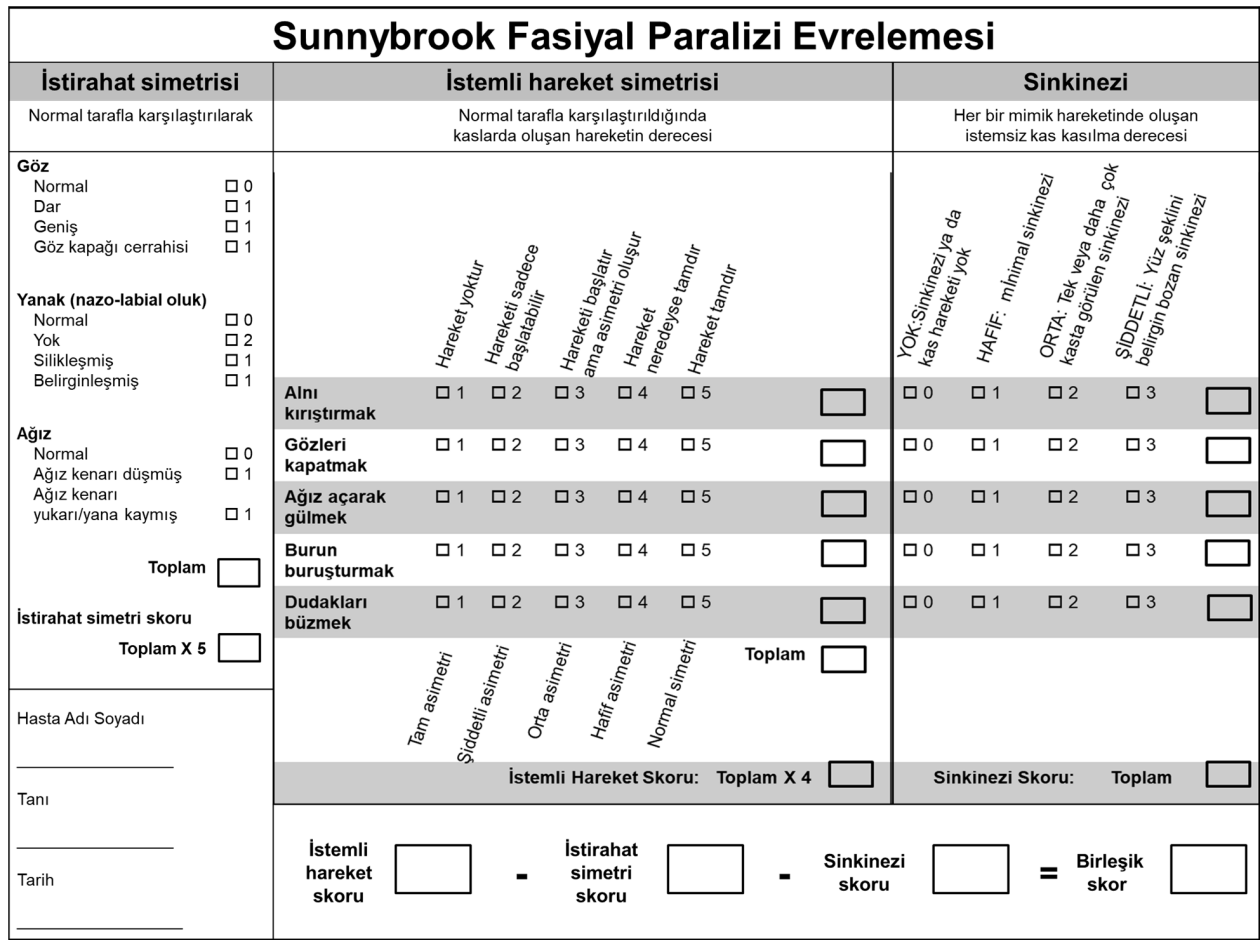
Turkish version of the Sunnybrook facial grading system.

### 2.2. Selection of the patients

Included in the study were 65 voluntary adult patients who had been diagnosed with unilateral PFP. After attaining consent from the patients, they were video recorded by the same researcher in a natural and luminous environment and asked to make facial movements according to the system requirements using a Sony ILCE-6000A camera. While recording was performed, the patients were requested to stay at rest, and then requested to perform the 5 standard facial movements (lifting the eyebrows, closing eyes gently, smiling mouth open, wrinkling the nose, and puckering the lips). All of the evaluations were performed based on these video recordings. 

### 2.3. Evaluation period

The group of evaluators was formed representing the scale users and consisted of researchers who had experience at different times in the field of otorhinolaryngology. Assigned to the study were 6 physicians, 4 of whom were specialists (2 professors and 2 assistant professors) and 2 of whom were residents. Before the evaluation stage, the SFGS and HBFGS forms were introduced to the researchers during a briefing, wherein each system was discussed in detail regarding the video recordings of 3 patients not included in the study by performing grading exercises.

In the SFGS, the 3 regions of the face, the eye, the cheek, and the mouth, were evaluated separately while the patient was at rest. On the other hand, the motor branches of the facial nerve were evaluated one-by-one during voluntary movements. Moreover, the presence of synkinesis was graded via the same voluntary movements. The composite score of the patient was calculated by subtracting the resting symmetry and synkinesis scores from the voluntary movement score. The SFGS was scored as 0 to 100 points and the score decreased as the severity of the disease increased. In the HBFGS, the patients were graded from 1 to 6, with a higher score indicating greater severity, which was contrary to the SFGS.

During data collection, the evaluators assessed the video recordings of the patients included in this study independently from each other. Evaluations were assessed in groups consisting of not more than 10 patients. In the first evaluation, both the SFGS and HBFGS forms were filled out for concurrent validity. While the researchers were allowed to pause and replay the video recordings during the evaluation, they were not permitted to go back and change the scores once the patients were scored. After the first evaluations were completed, evaluations with the SFGS were repeated 15 days later for the test/re-test period. In the second evaluation, the patients were assessed in a random order. 

### 2.4. Statistical analysis

Data were analyzed using SPSS v.24.0 software (IBM Corp., Armonk, NY, USA). Continuous variables were expressed as the mean ± standard deviation, median, minimum, and maximum values, whereas discrete variables were expressed as the number and percentage. The intraclass correlation coefficient (ICC) and the Cronbach’s alpha coefficient were used for examination of the inter- and intra-rater reliability. An ICC 95% confidence interval (95% CI) was also presented. Since synkinesis is not seen in the acute phase of PFP, the synkinesis scores of the SFGS were only evaluated in chronic PFP patients. In order to evaluate the concurrent validity, the Spearman correlation analysis was used in the examination of the numerical variables that were obtained in SFGS and HBFGS. In all of the analyses, P < 0.05 was accepted as statistically significant. The ICC values were interpreted, via the accepted criteria, where <0.4 = poor, 0.4–0.75 = fair to good, and ≥0.75 = excellent. Regarding the reliability of the scale, Cronbach’s alpha coefficient was determined as >0.7, indicating high internal consistency. Moreover, the generalizability (G) was checked as another indicator of reliability.

## 3. Results 

### 3.1. Patient population

Among the 65 patients, 33 were male (50.7%) and 32 were female (49.3%). The mean age of the patients was 45.06 (min-max: 17–73) years. Of the patients, 49 had acute PFP (≤3 months) and 16 had chronic PFP (>3 months). The most common PFP etiology was Bell’s palsy, with 45 cases (69.2%). It was followed by trauma in 7 patients (10.8%), Ramsay Hunt syndrome in 4 patients (6.2%), cholesteatoma in 3 patients (4.5%), acute otitis media in 2 patients (3.1%), parotid cancer in 2 patients (3.1%), and postoperative in 2 patients (3.1%). 

The mean SFGS composite score of the 65 patients was 44.98 ± 24.15 for the first assessment and 44.91 ± 24.30 for the second assessment. Table 1 summarizes the mean SFGS scores that were evaluated twice by the 6 evaluators.

**Table 1:** Average Sunnybrook system scores of the 65 patients evaluated twice by 6 evaluators.

	Assessment 1	Assessment 2
Resting symmetry		
Mean ± SD	9.61 ± 5.84	9.03 ± 6.19
Median (min–max)	10 (0–20)	10 (0–20)
Symmetry of voluntary movement		
Mean ± SD
55.08 ± 21.16	54.33 ± 21.48
Median (min–max)
56 (20–100)	56 (20–100)
Synkinesis		
Mean ± SD	0.44 ± 1.71	0.40 ± 1.77
Median (min–max)	0 (0–15)	0 (0–15)
Composite score		
Mean ± SD	44.98 ± 24.15	44.91 ± 24.30
Median (min–max)	46 (0–100)	45 (0–100)
SD = standard deviation.		

The mean HBFGS score of the 65 patients was 3.43 ± 1.32. According to the HBFGS, 31.8% of the patients were classified as grade II, 25.4% were grade III, 17.9% were grade IV, 16.9% were grade V, and 7.9% were grade VI. 

### 3.2. The reliability study 

For the inter-rater reliability, the ICC and Cronbach’s alpha coefficients were calculated for both measurements based on the obtained data from the 6 evaluators. The results are shown in Table 2. The ICC for resting symmetry, symmetry of voluntary movement, synkinesis, and the composite score, which are 4 components of the SFGS, were determined, respectively, as 0.822, 0.956, 0.606, and 0.957 for the first evaluation, and 0.805, 0.965, 0.594, and 0.965 for the second evaluation.

**Table 2:** Inter-rater reliability results of the Sunnybrook facial grading system.

	Assessment 1			Assessment 2		
	ICC	95% CI	Cronbach’s alfa	ICC	95% CI	Cronbach’s alfa
Resting symmetry	0.822	0.718–0.889	0.867	0.805	0.667–0.882	0.866
Eye	0.710	0.576–0.809	0.755	0.682	0.527–0.793	0.746
Cheek (naso-labial fold)	0.795	0.704–0.864	0.815	0.764	0.656–0.845	0.797
Mouth	0.788	0.685–0.863	0.824	0.805	0.701–0.876	0.847
Symmetry of voluntary movement	0.956	0.928–0.973	0.968	0.965	0.945–0.978	0.975
Brow lift	0.958	0.94–0.972	0.962	0.966	0.95–0.978	0.969
Gentle eye closure	0.942	0.911–0.963	0.953	0.95	0.918–0.969	0.963
Open mouth smile	0.925	0.878–0.954	0.945	0.932	0.891–0.957	0.947
Snarl	0.919	0.878–0.948	0.932	0.934	0.898–0.958	0.946
Lip pucker	0.902	0.85–0.938	0.921	0.924	0.897–0.953	0.942
Synkinesis*	0.606	0.118–0.887	0.664	0.594	0.091–0.837	0.645
Brow lift*	0.612	0.152–0.887	0.693	0.597	0.081–0.855	0.672
Gentle eye closure*	0.702	0.328–0.917	0.796	0.659	0.22–0.881	0.698
Open mouth smile*	0.367	–0.47–0.829	0.384	0.281	–0.503–0.740	0.338
Snarl *	0.475	–0.18–0.855	0.512	0.409	–0.243–0.830	0.467
Lip pucker*	0.589	0.111–0.891	0.603	0.537	0.203–0.821	0.586
Composite score	0.957	0.932–0.974	0.967	0.965	0.945–0.978	0.972

ICC = intraclass correlation coefficient; CI = confidence interval; * Synkinesis scores were evaluated only in chronic PFP patients.

For the intra-rater reliability, the results of assessments that were performed over 2 different time spans by the 6 evaluators were compared. Table 3 shows the ICC and Cronbach’s alpha coefficients of the composite scores, which were calculated separately based on the data from each evaluator. Based on the ICC results, in the resting symmetry score, 3 evaluators reported excellent correlation, whereas the other 3 evaluators reported good correlation. In the symmetry of voluntary movement score, all of the evaluators reported excellent correlation. In the synkinesis score, 3 evaluators reported excellent correlation, while 1 evaluator reported good correlation and 2 evaluators reported no correlation. In the composite score, excellent correlation was determined by all of the evaluators. In the analysis of the averages of the 4 components of the SFGS for the intra-rater reliability, the ICC results were determined as 0.842, 0.956, 0.794, and 0.937, while the Cronbach’s alpha results were determined as 0.809, 0.956, 0.792, and 0.948, respectively. 

**Table 3:** Intra-rater reliability results of the composite score of the Sunnybrook facial grading system.

	ICC	95% CI	Cronbach’s alfa
Evaluator 1	0.892	0.818–0.935	0.899
Evaluator 2	0.958	0.931–0.974	0.958
Evaluator 3	0.938	0.89–0.964	0.944
Evaluator 4	0.973	0.955–0.983	0.972
Evaluator 5	0.958	0.925–0.976	0.962
Evaluator 6	0.933	0.891–0.959	0.934
Evaluators 1–6*	0.937	0.958–0.948	0.948

ICC = intraclass correlation coefficient; CI = confidence interval; * = average of all measurements.

The G theory is another indicator of reliability for a system. In G, all of the potential sources of error in the measurement were assessed and the percentages of the explanations of the total variance by the obtained results were examined. The G coefficient obtained from these variance values was determined as G = 0.772. This result showed that the result of the SFGS was free of potential fault factors. 

### 3.3. The validity study 

For the concurrent validity, the results of the test were compared with a concurrently administered tool whose psychometric studies had already been performed and the correlation was checked between the 2 results. In this study, the correlation between the SFGS and HBFGS were investigated and a statistically significant strong negative correlation was detected, by all of the evaluators (Table 4). In the SFGS, the scores decreased as the severity of the disease increased, whereas in the HBFGS, the scores increased as the severity of the disease increased. For this reason, a negative correlation was observed. 

**Table 4:** Correlation between the Sunnybrook and House- Brackman facial grading systems.

Evaluator 1	P < 0.01; r = –0.847
Evaluator 2	P < 0.01; r = –0.913
Evaluator 3	P < 0.01; r = –0.907
Evaluator 4	P < 0.01; r = –0.939
Evaluator 5	P < 0.01; r = –0.862
Evaluator 6	P < 0.01; r = –0.884

Statistical significance was accepted as P < 0.05; Spearman correlation analysis.

## 4. Discussion 

Whether a scale is suitable for its purpose or not is investigated via validity and reliability studies [6]. When a questionnaire is translated into another language, a validation study should be performed. This process has 3 stages. These are linguistic validity, reliability validity, and subject validity studies. Linguistic validity studies provide the same meaning for everybody. These scales should also be reliable. The reports of the different physicians at different times for different patients must have comparable consistency. Hence, it is expected that the inter- and intra-rater reliability should be high. Validity is the display of whether the system is goal-oriented or not.

In an ideal facial paralysis grading system, it has been suggested that: 1) facial functions are able to be scored regionally, 2) both static and dynamic measurements can be performed, 3) it can examine facial palsy sequels, 4) the inter- and intra-rater reliability is high, 5) it is sensitive to changes that occur over time, and 6) it is convenient for clinical use [5,7–9]. There is no doubt that the HBFGS, which is used most commonly, is quite practical and convenient for clinical use. However, its poor regional scoring and presence of several facial movements in the same grade make its inter-rater reliability low, especially in grades II and IV. When considering the alternatives, resting symmetry in the Sydney facial grading system [10] and facial palsy sequels in the Yanagihara facial grading system [11] was not included in the evaluation. The SFGS is the only system that meets all of the suggested criteria [5]. For this reason, it was aimed to translate the SFGS into Turkish in the current study.

Translation problems are encountered naturally in the process of adaptation of the systems to different languages. There may not be a counterpart of a word in another language or its counterpart may be insufficient to express the desired meaning exactly. The same problem was encountered for the word snarl in the linguistic validity process. It was observed that it was not understood correctly by Turkish patients. For this reason, the term wrinkle the nose was used for the word snarl, and it was observed that the Turkish patients understood this term better.

Each evaluator should come up with a similar result with an evaluation performed using a standard tool. For this reason, agreement between the evaluators should be high in facial grading systems. At the same time, the results obtained from a reliable system should be repeatable. In other words, the evaluations performed at different times by the same physician should be compatible with each other. It was reported that the SFGS was a reliable and valid scale in previously performed validity studies [12–16]. In Table 5, the inter- and intra-rater reliability results are compared with the results of the validity studies in the different languages in the literature. In the current study, it was found that the inter-rater reliability ICC results were 0.957 and 0.965 for the first and the second assessment, respectively, and the intra-rater reliability ICC results were between 0.892 and 0.973 for the Turkish version of the SFGS. When these results were examined, it was observed that the Turkish SFGS had the same measurement properties as the original scale and was reliable in terms of both repeatability and agreement.

**Table 5:** Inter- and intra-rater reliability results of the reported validation studies of the Sunnybrook facial grading system in the different languages in the literature.

	Number ofraters	Number of patients	Inter-rater ICC(measurement 1–2)	Intra-rater ICC(min-max)
Hu et al.12	8	22	0.982–0.970	0.839–0.929
Kanerva et al.13	26	8	0.997–0.997	0.864–0.995
Neely et al.14	2	30	0.890	0.948–0.970
Pavese et al.15 (Italian version)	6	29	0.93–0.98	0.97–0.98
Neumann et al.16 (German version)	5	18	0.918–0.940	0.668–0.974

ICC = intraclass correlation coefficient.

When the components of the SFGS were examined in the current study, the most compatible scores were observed in voluntary movement, whereas the least compatible scores were observed in synkinesis. Synkinesis, as is known, occurs 12 to 18 months after the onset of facial paralysis. Otorhinolaryngologists examine facial palsy patients more often in the acute phase. Relatively low synkinesis scores have been related to otorhinolaryngologists not being in the habit of checking this parameter. Similarly, both Kayhan et al. [17] and Coulson et al. [10] reported the lowest reliability in synkinesis scores. 

Another reliability criterion used in this study was G, which is a statistical theory that allows the assessment of behavioral reliability, to design and examine reliable observations, and is based on variance analysis. The G value obtained in this study showed that the Turkish version of the SFGS was free of potential mistakes and only evaluated facial paralysis in the patients. 

In the process of validation, the validity of the system should be displayed as well. Validity displays whether the system serves the desired purpose or not. The comparison of the new system results with another system, which is the same goal-oriented, widely accepted, and standard, is a convenient approach [6]. Kanerva et al. compared the SFGS and HBFGS and reported that the inter-rater reliability was higher in the SFGS. Coulson et al. compared the SFGS, HBFGS, and Sydney facial grading systems and reported high compatibility, especially in the voluntary movement scores [10]. When the results of the SFGS combined score and the HBFGS were compared herein, strong correlation was determined between the 2 systems. This result showed that the Turkish version of the SFGS had concurrent validity. 

The main limitation of this study was the use of the HBFGS instead of the Facial nerve grading system 2.0 (FNGS 2.0) for concurrent validity. In 2009, the Facial Nerve Disorders Committee designed the FNGS 2.0 to overcome the criticism of the HBFGS and recommended the use of this new system [18]. However, to date, no validated Turkish version of the FNGS 2.0 has been developed. Additionally, the best known and most widely used system of grading facial paralysis is still the HBFGS. Due to the abovementioned reasons, the HBFGS was chosen to evaluate concurrent validity in the current study.

In conclusion, the SFGS is becoming more commonly used worldwide because it meets all of the necessary criteria that should be found in an ideal facial grading system. The present study translated the SFGS into Turkish and demonstrated that this new version of the scale was valid and reliable. The Turkish version of the SFGS, which was formed at the end of this study, can be used confidently for the evaluation, follow-up, and reporting of patients with facial nerve disorders.

## Acknowledgments

The authors declare that this study received no financial support. Ethics committee approval was received for this study from the Ethics Committee of Pamukkale University (60116787-020/4322). Written informed consent was obtained from the patients who participated in this study.
